# Computed tomography angiography versus Agatston score for diagnosis of coronary artery disease in patients with stable chest pain: individual patient data meta-analysis of the international COME-CCT Consortium

**DOI:** 10.1007/s00330-022-08619-4

**Published:** 2022-03-10

**Authors:** Viktoria Wieske, Mario Walther, Benjamin Dubourg, Hatem Alkadhi, Bjarne L. Nørgaard, Matthijs F. L. Meijs, Axel C. P. Diederichsen, Yung-Liang Wan, Hans Mickley, Konstantin Nikolaou, Abbas A. Shabestari, Bjørn A. Halvorsen, Eugenio Martuscelli, Kai Sun, Bernhard A. Herzog, Roy P. Marcus, Sebastian Leschka, Mario J. Garcia, Kristian A. Ovrehus, Juhani Knuuti, Vladymir Mendoza-Rodriguez, Nuno Bettencourt, Simone Muraglia, Ronny R. Buechel, Philipp A. Kaufmann, Elke Zimmermann, Jean-Claude Tardif, Matthew J. Budoff, Peter Schlattmann, Marc Dewey, on behalf of the COME-CCT Consortium

**Affiliations:** 1 Department of Radiology, Charité - Universitätsmedizin Berlin, Charitéplatz 1, 10117 Berlin, Germany; 2 Department of Fundamental Sciences, Jena University of Applied Sciences, Jena, Germany; 3 Cardiac Imaging Unit, Department of Radiology, Rouen University Hospital, Rouen, France; 4 Institute of Diagnostic and Interventional Radiology, University Hospital Zurich, Zurich, Switzerland; 5 Department of Cardiology, Aarhus University Hospital, Aarhus, Denmark; 6 Department of Cardiology, University Medical Centre Utrecht, Utrecht, Netherlands; 7 Department of Cardiology, Odense University Hospital, Odense, Denmark; 8 Medical Imaging and Radiological Sciences, College of Medicine, Chang Gung University, Chang Gung Memorial Hospital at Linkou, Taoyaun City, Taiwan; 9 Department of Diagnostic and Interventional Radiology, University Hospital of Tübingen, Tübingen, Germany; 10 Modarres Hospital, Shahid Beheshti University of Medical Sciences, Tehran, Iran; 11 Department of Cardiology, Ostfold Hospital Trust, Grålum, Norway; 12 Department of Internal Medicine, University of Rome Tor Vergata, Rome, Italy; 13 Department of Radiology, Baotou Central Hospital, Baotou, Inner Mongolia Province China; 14 HeartClinic Lucerne, Lucerne, Switzerland; 15 Cantonal Hospital of Lucerne, Lucerne, Switzerland; 16 Department of Radiology, Kantonsspital St Gallen, St Gallen, Switzerland; 17 Department of Cardiology, Montefiore, University Hospital for the Albert Einstein College of Medicine, New York, NY USA; 18 Turku University Hospital and University of Turku, Turku, Finland; 19 Department of Cardiology, National Institute of Cardiology and Cardiovascular Surgery, Havana, Cuba; 20 Department of Cardiology, Centro Hospitalar de Vila Nova de Gaia, Vila Nova de Gaia, Portugal; 21 Department of Cardiology, S Chiara Hospital, Trento, Italy; 22 Department of Nuclear Medicine, University Hospital Zurich, Zurich, Switzerland; 23 Montreal Heart Institute, Université de Montréal, Montréal, Canada; 24 Los Angeles Biomedical Research Institute, Torrance, CA USA; 25 Institute of Medical Statistics, Computer Sciences and Data Science, University Hospital of Friedrich Schiller University Jena, Jena, Germany

**Keywords:** Computed tomography angiography, Coronary angiography, Coronary artery disease

## Abstract

**Objectives:**

There is conflicting evidence about the comparative diagnostic accuracy of the Agatston score versus computed tomography angiography (CTA) in patients with suspected obstructive coronary artery disease (CAD).

**Purpose:**

To determine whether CTA is superior to the Agatston score in the diagnosis of CAD.

**Methods:**

In total 2452 patients with stable chest pain and a clinical indication for invasive coronary angiography (ICA) for suspected CAD were included by the Collaborative Meta-analysis of Cardiac CT (COME-CCT) Consortium. An Agatston score of > 400 was considered positive, and obstructive CAD defined as at least 50% coronary diameter stenosis on ICA was used as the reference standard.

**Results:**

Obstructive CAD was diagnosed in 44.9% of patients (1100/2452). The median Agatston score was 74. Diagnostic accuracy of CTA for the detection of obstructive CAD (81.1%, 95% confidence interval [CI]: 77.5 to 84.1%) was significantly higher than that of the Agatston score (68.8%, 95% CI: 64.2 to 73.1%, *p* < 0.001). Among patients with an Agatston score of zero, 17% (101/600) had obstructive CAD. Diagnostic accuracy of CTA was not significantly different in patients with low to intermediate (1 to < 100, 100–400) versus moderate to high Agatston scores (401–1000, > 1000).

**Conclusions:**

Results in our international cohort show CTA to have significantly higher diagnostic accuracy than the Agatston score in patients with stable chest pain, suspected CAD, and a clinical indication for ICA. Diagnostic performance of CTA is not affected by a higher Agatston score while an Agatston score of zero does not reliably exclude obstructive CAD.

**Key Points:**

*• CTA showed significantly higher diagnostic accuracy (81.1%, 95% confidence interval [CI]: 77.5 to 84.1%) for diagnosis of coronary artery disease when compared to the Agatston score (68.8%, 95% CI: 64.2 to 73.1%, p < 0.001).*

*• Diagnostic performance of CTA was not affected by increased amount of calcium and was not significantly different in patients with low to intermediate (1 to <100, 100–400) versus moderate to high Agatston scores (401–1000, > 1000).*

*• Seventeen percent of patients with an Agatston score of zero showed obstructive coronary artery disease by invasive angiography showing absence of coronary artery calcium cannot reliably exclude coronary artery disease.*

**Supplementary Information:**

The online version contains supplementary material available at 10.1007/s00330-022-08619-4.

## Introduction

Coronary artery calcium (CAC) has become an independent diagnostic marker besides traditional risk factors of atherosclerosis for the detection of coronary artery disease (CAD) [1] and is most commonly quantified using the Agatston score method [2]. CAC quantification is frequently used in asymptomatic patients for risk stratification. While there is evidence that traditional risk factors are associated with an increase in arterial calcification [3], others report on a discrepancy between traditional risk factors and coronary artery calcification when countries are downgraded as low-risk countries. Diederichsen et al showed through the example of Germany and Denmark using low-risk models, that individuals with severe coronary atherosclerosis are more commonly assigned to low to intermediate pre-test probability of CAD [4]. The absence of CAC is associated with low rates of cardiovascular events such as myocardial infarction, cardiovascular death, or unstable angina pectoris in asymptomatic individuals [5]. Evaluation of CAC as an independent parameter for the detection of obstructive CAD has yielded inconsistent results. In contrast, the absence of coronary artery calcium does not reliably exclude CAD based on non-calcified plaque lesions [6, 7].

The CORE-64 international multicentre study showed computed tomography angiography (CTA) to have significantly lower diagnostic accuracy in patients with CAC elevated above 600 [8]. As commonly known, CTA shows good diagnostic accuracy in detecting obstructive CAD in patients with stable chest pain [9, 10].

Overall, there is conflicting evidence about the comparative diagnostic accuracy of the Agatston score versus CTA, and thus, the diagnostic role of the Agatston score in detecting obstructive CAD is still unclear. Therefore, different international guidelines call for more scientific evidence from large cohorts to reliably decide how to use the Agatston score in patients with stable chest pain [6, 11, 12].

We sought to collaboratively determine and compare the diagnostic accuracy of CTA and the Agatston score in detecting obstructive CAD in stable chest pain patients with a clinical indication for invasive coronary angiography (ICA) by using individual patient data (IPD) from the worldwide Collaborative Meta-Analysis of Cardiac CT (COME-CCT) Consortium. Furthermore, we investigated if the diagnostic accuracy of CTA was affected by higher Agatston scores.

## Methods

### Patients

**Fig. 1:**
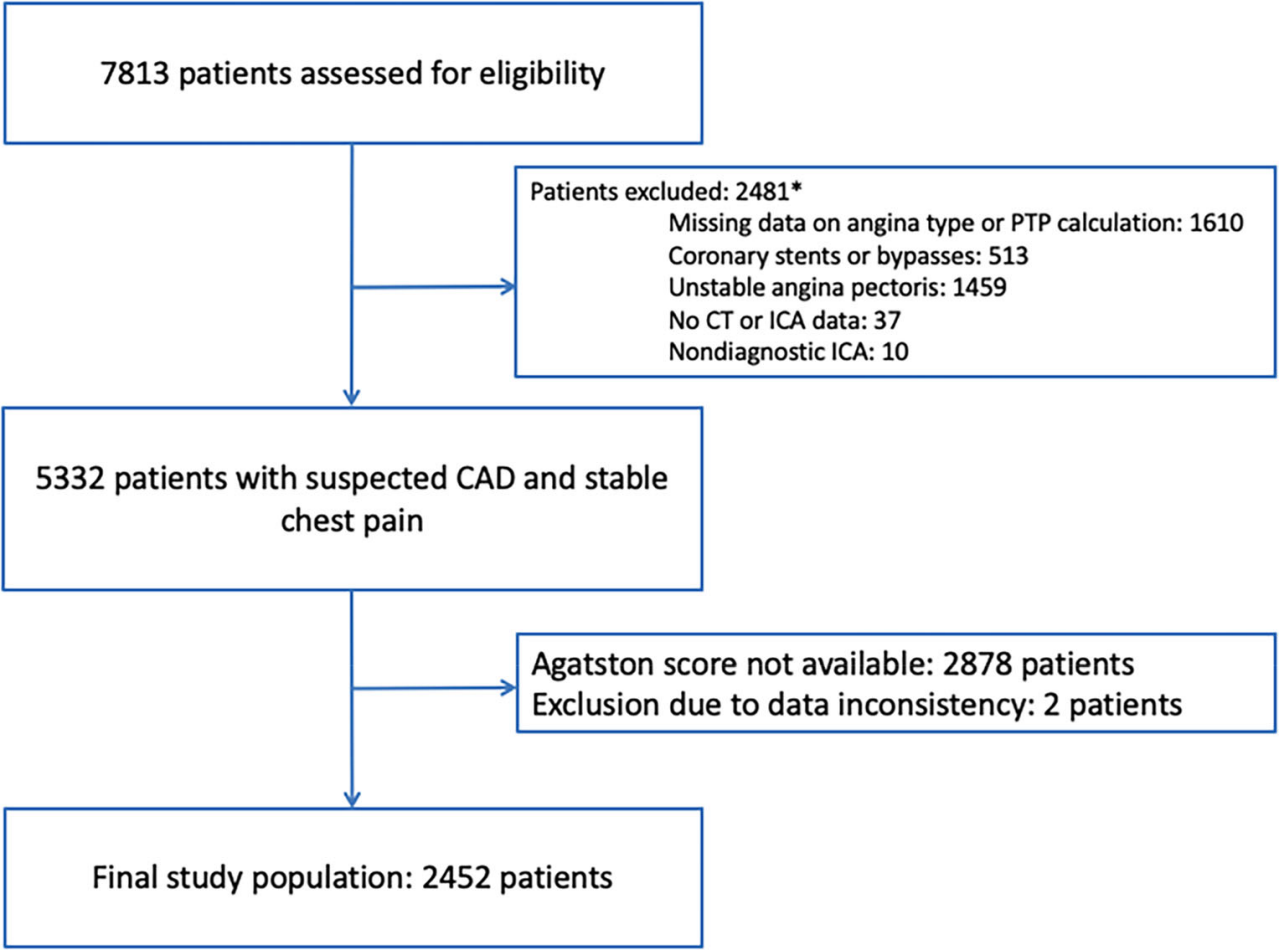
Flow of study patients. Patients with unstable presentation, bypass grafts and/or coronary stents, nondiagnostic ICA, or no CT/ICA data were excluded as previously defined and shown [10, 13]. Patients who underwent CTA without Agatston score calculation or data inconsistencies were excluded after contacting responsible authors. *Multiple reasons per patient possible. *PTP*, pre-test probability; *CT*, computed tomography; *ICA*, invasive coronary angiography; *CAD*, coronary artery disease

Patients with a clinical indication for ICA, who were also prospectively enrolled to undergo cardiac CT, from the COME-CCT provided the basis for our comparison of the diagnostic accuracy of the Agatston score versus that of CTA with ICA as the reference standard using individual patient data [13]. Patients with unstable presentation, such as known CAD, coronary stents, or bypass grafts, were excluded (Fig. 1).

The international COME-CCT Consortium consists of 76 worldwide studies with a total of 7813 individual patient datasets. Eligible studies were identified by the meta-analysis of Schuetz et al [9], and additional unpublished search updates were performed to ensure inclusion of the most recent studies. Participating partners were asked to provide detailed information on a per-patient basis as follows: patient characteristics, technical information, procedure and results of CTA and ICA, risk factors, and additional tests. All included patients prospectively underwent both tests, ICA and CTA, on CT scanners with at least 12 rows, and obstructive CAD was defined as ≥ 50% diameter stenosis by ICA. Details of enrolment and methods are available described elsewhere [13], and the study was registered in the PROSPERO Database for Systematic Reviews (CRD42012002780). The current analysis included all patients from the main collaborative analysis cohort with information on the Agatston score [10] and was a prospectively defined outcome measure of the COME-CCT Consortium [13].

### CTA and Agatston scores

In this analysis, we used the Agatston score for CAC quantification [2] using unenhanced images obtained before the CTA scan. CTA was read by experienced investigators as part of prospective study protocols on workstations enabling two- and three-dimensional postprocessing of CTA datasets, and at least 50% coronary diameter stenosis was considered to represent obstructive CAD. Agatston scores of > 400 were considered positive for obstructive CAD [11, 14, 15]. Following Agatston et al, any value below 1.0 was classified as an Agatston score of zero. The ranges for scores of 1 to < 100, 100 to 400, 401 to 1000, and above 1000 were defined as follows: between 1.0 and below 100.0, exactly 100.0 to less than or equal to 400.0, larger than 400.0 to less than or equal to 1000.0, and larger than 1000.0. Pre-test probability (PTP) was calculated as previously described [10] using information on sex, age, and angina pectoris type on the patient level, and categorized into low (< 15%), intermediate (15 – 65%), and high (> 65%). PTP was not a pre-defined inclusion criterion of the COME-CCT Consortium, but was assessed as a mandatory item for analysis on the per patient level in all included patients in this sub-analysis of the COME-CCT Consortium.

### ICA

Obstructive CAD was defined as a diameter reduction of at least 50% of the coronary artery by ICA as the reference standard and was analyzed on the patient level. ICA was performed according to local standards and was available in all included patients.

### Statistical analysis

All results are reported as mean ± standard deviation (SD) for quantitative data with normal distribution, median with interquartile range (IQR) for ordinal or not normally distributed data, and proportion of 95% confidence interval (CI) for categorical data. Nondiagnostic CTA results were implemented in a worst-case scenario using an intention-to-diagnose approach by counting CTA results as positive when ICA was negative and as negative when ICA was positive (treating nondiagnostic CTA cases as if they were false positive or false negative by ICA result, respectively). Diagnostic performance (in terms of diagnostic accuracy defined as the proportion of correct test results, sensitivity, specificity, and predictive values) was calculated and compared between both tests versus the reference standard ICA.

For statistical comparison of diagnostic performance of CTA with Agatston score results, we applied a logistic regression model with random effects [16] to account for dependencies of the two diagnostic tests within patients and for study-specific clustering [17–19].

Overall diagnostic accuracies (percentage and 95% CI) of CTA in patients with different Agatston score groups, zero Agatston score, low (1 to < 100), intermediate (100 to 400), moderately (401 to 1000), and highly increased (above 1000) Agatston score, were calculated on the per-patient level. Calculations were done using exact binomial distribution. Statistical significance was assumed for *p* ≤ 0.05. Statistical analyses were performed using SAS (version 9.4).

## Results

**Fig. 2:**
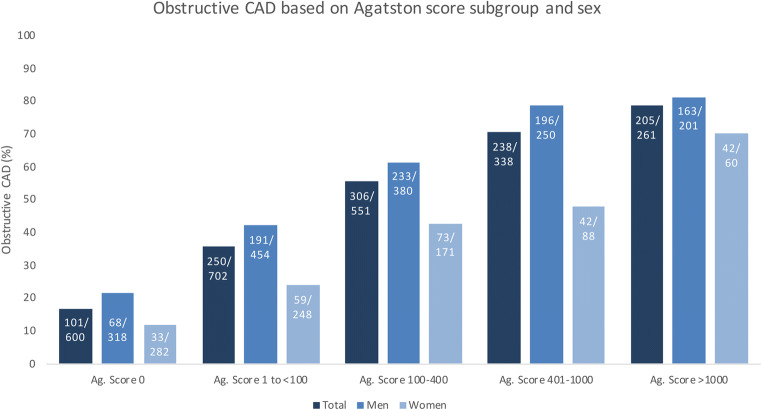
Obstructive CAD by Agatston score subgroup and sex. 1100 of 2452 included patients were diagnosed with obstructive CAD. Proportion of obstructive CAD based on subgroup of zero, low, intermediate, moderately and highly increased Agatston score and gender is shown as percentages. Absolute numbers referring to respective subgroups are presented within each bar. In all subgroups, obstructive CAD was more frequent in men. Ag. Score, Agatston score

A total of 2452 stable chest pain patients with suspected CAD and an indication for ICA were included (Fig. 1) in this collaborative meta-analysis of the COME-CCT Consortium comparing CTA findings with Agatston scores for CAD diagnosis based on both published and unpublished results of 28 studies (from 29 original datasets as two studies were combined due to small sample size of each subset) conducted in 14 countries [20–46]. The median Agatston score in the total of 2452 patients was 74.1 with an IQR of 1 to 389. Obstructive CAD by ICA was diagnosed in 45% of patients (1100 of 2452, Table 1, Fig. 2). About two-thirds of the patients were male (1603 of 2452); mean age of included patients was 62 ± 10 years; and half of the patients had arterial hypertension (56%) and hyperlipidemia (52%; Table 1, detailed characteristics by Agatston score subgroup are provided in Appendix Table 4 and Appendix Table 5). PTP calculation identified twelve patients (0.49%) with low PTP, 1884 patients (76.77%) with intermediate PTP, and 558 with high PTP (22.74%). In the 2452 patients suitable for intraindividual patient-based analysis of CTA findings and Agatston scores, CT was performed in 334, 660, 1202, 227, and 29 patients on CT scanners with 16 rows, 32 rows, 64 rows, 128 rows, and 320 rows, respectively.

**Table 1:** Characteristics of the 2452 patients with stable chest pain*

Characteristics	*n* = 2452
Age, y	62 ± 10
Male sex, *n* (%)	1603 (65.4)
Agatston score	
Median	74.1
Range (minimum to maximum)	0 to 6209.6
IQR	1 to 389
Arterial hypertension, *n* (%)^§^	1332/2381 (55.9)
Diabetes mellitus, *n* (%)^§^	442/2395 (18.5)
Hyperlipidaemia, *n* (%)^§^	1172/2273 (51.6)
Current smoker, *n* (%)^§^	708/2396 (29.5)
Body mass index^†^	26.8 ± 4.1
Prevalence of obstructive CAD on ICA	1100 (44.9)
Angina pectoris classification, *n* (%)	
Typical angina	1078 (44.0)
Atypical angina	746 (30.4)
Nonanginal chest pain	467 (19.0)
Other chest discomfort	161 (6.6)
Pre-test probability, %	49 ± 17

*Plus-minus values are means ± SD unless otherwise stated. Percentages are based on analysis of all 2452 patients unless otherwise stated. ^§^Missing information as follows per category, *n* (%): hypertension 71 (2.9%), diabetes mellitus 57 (2.3%), hyperlipidaemia 179 (7.3%), current smoker 56 (2.3%). ^†^Calculated as the weight in kilogrammes divided by the square of the height in metres; body mass index calculation based on 2425 patients (1.1% missing information on BMI). *CAD* coronary artery disease, *ICA* invasive coronary angiography

### Diagnostic performance of CTA and Agatston score

**Fig. 3:**
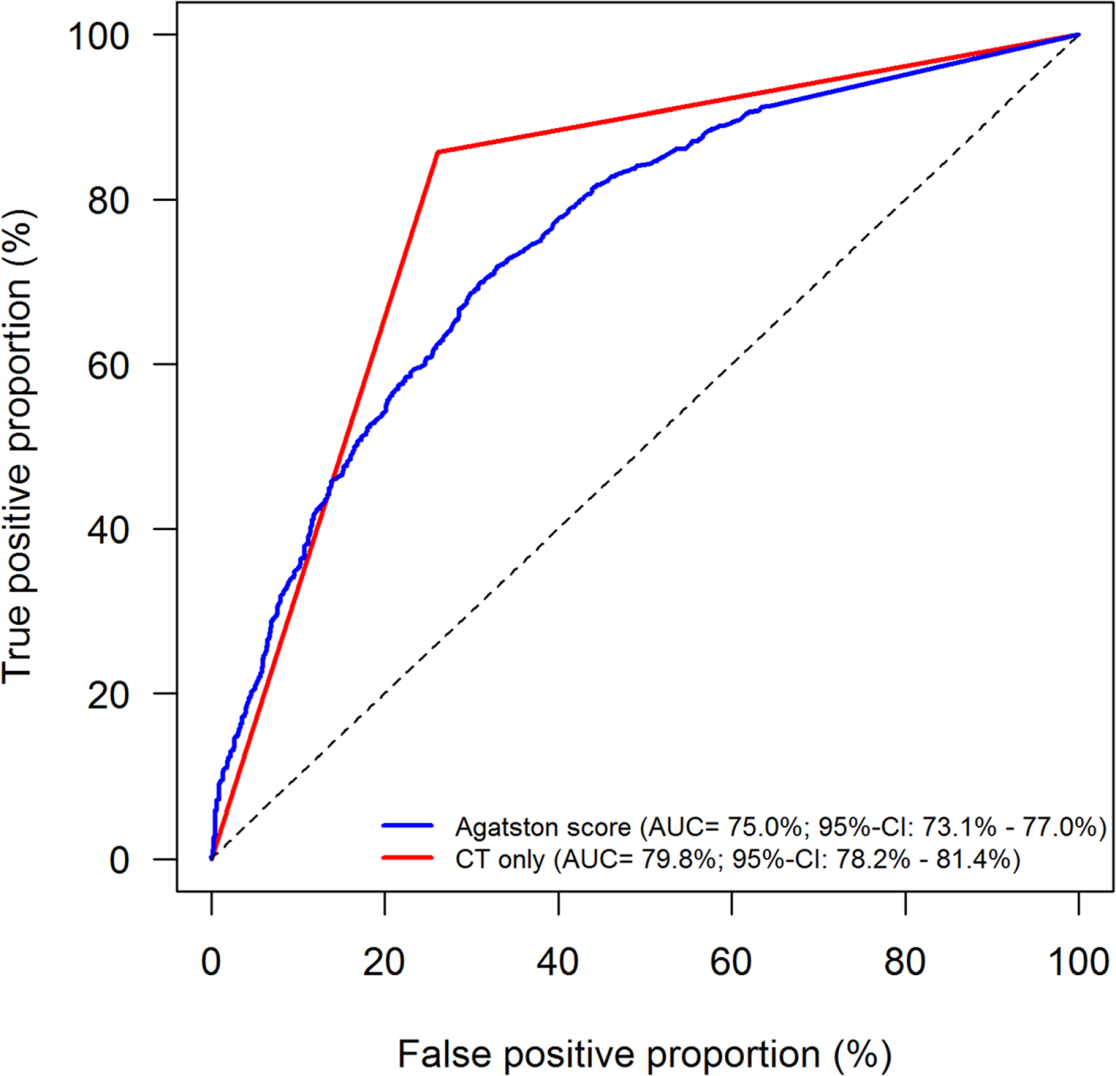
Area under the receiver operating characteristic curves of CTA and Agatston score. Receiver operating characteristic curves of CTA versus Agatston score for diagnosis of CAD in all 2452 patients showing better overall performance for CTA. AUC, area under the curve; CT, computed tomography

**Fig. 4:**
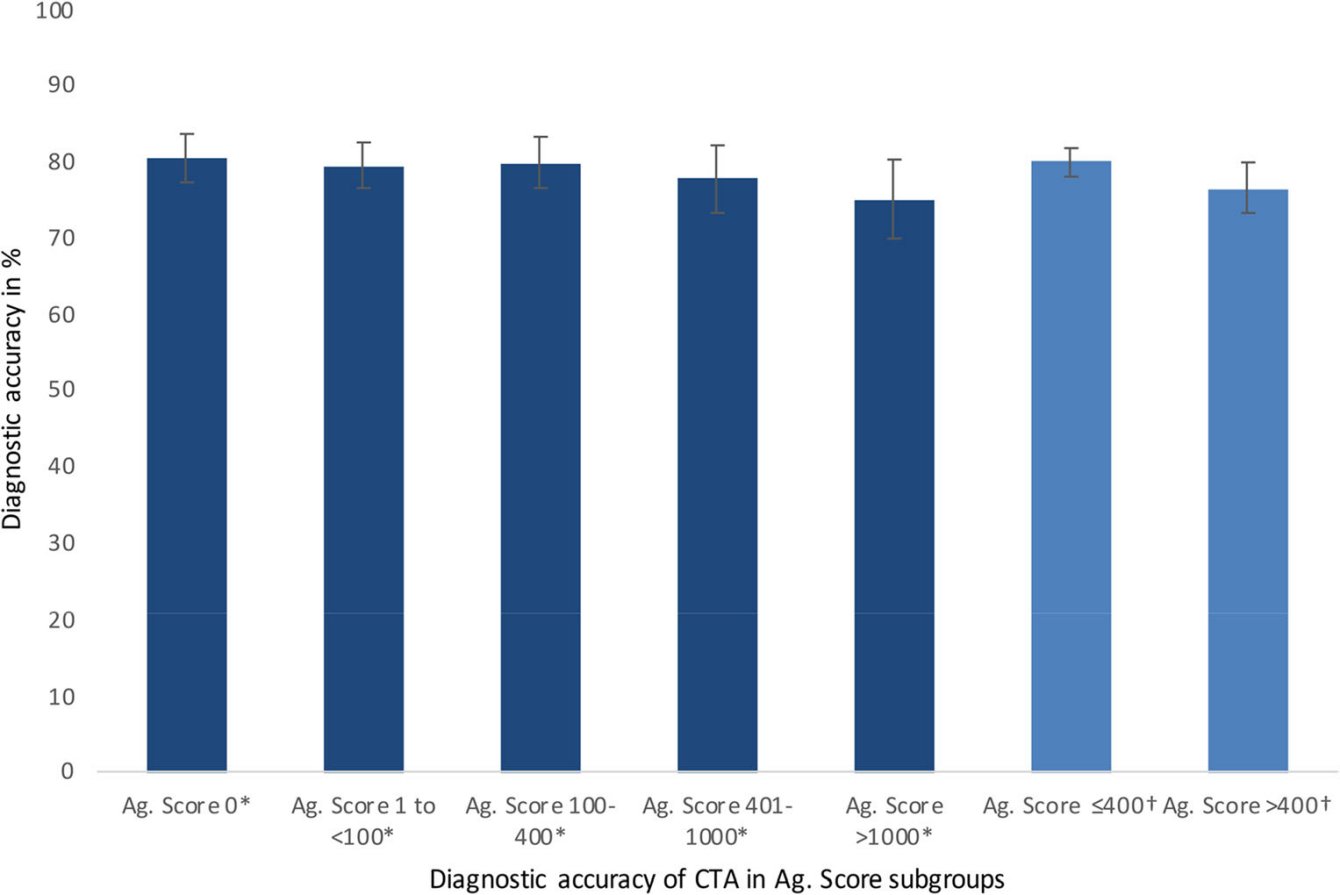
Similar diagnostic accuracy of CTA in patients with low, intermediate, moderately, and highly increased Agatston scores. Error bars based on 95% CI. *Diagnostic accuracy of CTA was not significantly different in Agatston score subgroups. † Equally, additional analysis in patients with an Agatston score of ≤ 400 versus > 400 showing diagn ostic accuracy of CTA not significantly different in both groups. Ag. Score, Agatston score; CTA, computed tomography angiography

**Fig. 5:**
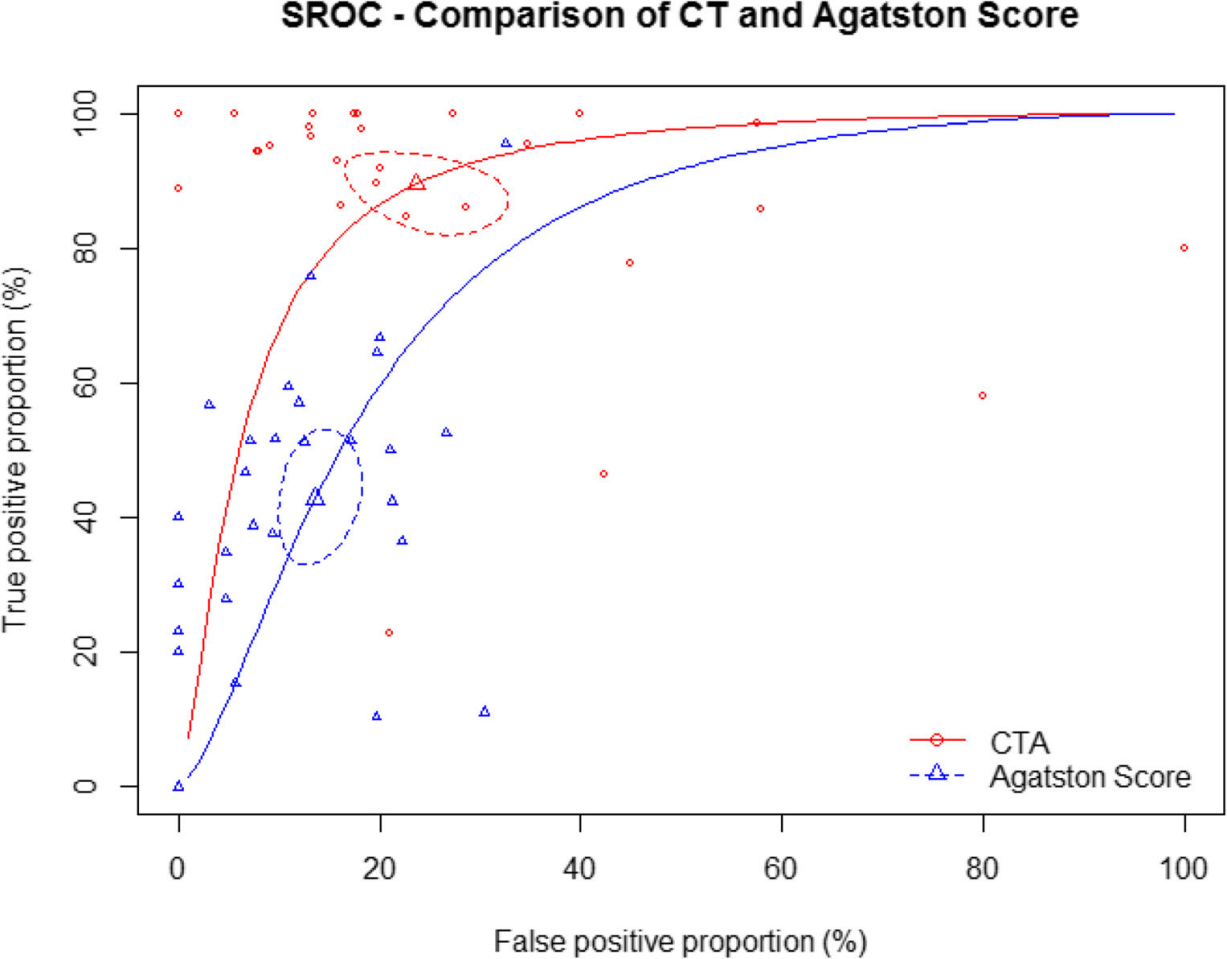
Summary receiver operating characteristic curve (SROC) for CTA and Agatston score

The cross-tabulation of CTA and Agatston score versus the reference standard ICA and the cross-tabulation of CTA versus Agatston score are shown in Tables 2 and 3, respectively. Nondiagnostic CT examinations occurred in 11.2% of patients and were implemented in an intention-to-diagnose approach as previously described; detailed results for diagnostic performance excluding nondiagnostic examinations are presented in the Supplement (Appendix Table 1-3). Diagnostic accuracy of CTA was significantly higher than that of the Agatston score for the detection of obstructive CAD (81.1%, 95% CI: 77.5 to 84.1% versus 68.8%, 95% CI: 64.2 to 73.1%, *p* < 0.0001, Table 4) with a positive likelihood ratio of 3.3 versus 3.5 and a negative likelihood ratio of 0.2 versus 0.7. The sensitivity of an Agatston score above 400 for identifying patients with CAD (40.9%, 95% CI: 36.6 to 45.3%) was significantly lower than that of CTA (86.4%, 95% CI: 83.7 to 88.7%, *p* < 0.0001). The specificity of the Agatston score above 400 was significantly higher compared with CTA (88.2%, 95% CI: 85.8 to 90.2% versus 73.2%, 95% CI: 69.5 to 76.6%, *p* < 0.0001, Table 4). The area under the receiver operating characteristics curve for CTA was larger (79.8%, 95% CI: 78.2 to 81.4%) compared to that for the Agatston score (75.0%, 95% CI: 73.1 to 77.0%, Fig. 3). Summary receiver operating characteristics curve (SROC) and study-specific forest plots are shown in Figs 4 and 5, respectively. Funnel plots for CTA and Agatston score can be found in the Supplement (Appendix Figure 1A, Appendix Figure 1B, respectively). The negative predictive value of CTA (85.2%, 95% CI: 81.3 to 88.3%) was significantly higher than that of the Agatston score (64.1%, 95% CI: 58.4 to 69.4%, *p* < 0.0001). Most importantly, among the 1100 patients with obstructive CAD on ICA, 657 showed a false negative Agatston score of up to 400 whereas only 157 were false negative on CTA (Table 2).

**Table 2:** Direct comparison of CTA and Agatston score versus the reference standard of invasive coronary angiography according to STARD (47)

	Invasive coronary angiography	
	Positive	Negative
CTA		
Positive	943 (85.7%)	353 (26.1%)
Negative	157 (14.3%)	999 (73.9%)
Total	1100 (100.0%)	1352 (100.0%)
Agatston score*		
Positive	443 (40.3%)	156 (11.5%)
Negative	657 (59.7%)	1196 (88.5%)
Total	1100 (100.0%)	1352 (100.0%)

*An Agatston score above 400 was considered positive. *STARD* Standards for Reporting of Diagnostic Accuracy, *CTA* computed tomography angiography

**Table 3:** 2-by-2 table of CTA and Agatston score according to STARD (47)*

	CTA	
	Positive	Negative
Agatston score^§^		
Positive	447 (18.2%)	152 (6.2%)
Negative	849 (34.6%)	1004 (41.0%)

*Percentages are based on 2452 patients. *STARD* Standards for Reporting of Diagnostic Accuracy, *CTA* computed tomography angiography. ^§^An Agatston score above 400 was considered positive

**Table 4:** Diagnostic performance of CTA and Agatston score on the patient level*

	CTA *n*/total *n* (% [95% CI*])	Agatston score^§^ *n*/total *n* (% [95% CI*])	*p*-value*
Diagnostic accuracy	1942/2452 (81.1% [77.5 – 84.1])	1639/2452 (68.8% [64.2–73.1])	< 0.0001
Sensitivity	943/1100 (86.4% [83.7 – 88.7])	443/1100 (40.9% [36.6–45.3])	< 0.0001
Specificity	999/1352 (73.2% [69.5 – 76.6])	1196/1352 (88.2% [85.8–90.2])	< 0.0001
Negative predictive value	999/1156 (85.2% [81.3 – 88.3])	1196/1853 (64.1% [58.4–69.4])	< 0.0001
Positive predictive value	943/1296 (73.1% [67.9 – 77.8])	443/599 (75.8% [70.2–80.7])	0.2206
Positive likelihood ratio	3.54 [2.61–4.81]	3.10 [2.37–4.06]	
Negative likelihood ratio	0.14 [0.09–0.23]	0.67 [0.60–0.75]	
DOR	31.42 [13.68–72.14]	5.40 [3.60–8.09]	

*CI, confidence interval. Estimates, 95% CI, and *p*-values are based on a model with study-specific random intercept taking test correlation within patients into account. ^§^An Agatston score above 400 was considered positive

Positive likelihood ratio, negative likelihood ratio, and diagnostic odds ratio (DOR) based on random effect models for each test

### Zero Agatston score

A total of 600 patients had a zero Agatston score. Diagnostic accuracy of CTA in this subgroup was 80.7% (95% CI: 77.5 to 83.8%) with a negative predictive value of 95.5% (95% CI: 93.5 to 97.5%) and positive predictive value of 45.8% (95% CI: 38.5 to 53.1%). Pre-test probability in this subgroup was 42% (± 16%).

16.8% of patients with a zero Agatston score had obstructive CAD by ICA (101 of 600), whereas only 3.2% of those were false negative by CTA (19 of 600).

### CTA accuracy in patients with higher Agatston scores

**Fig. 6:**
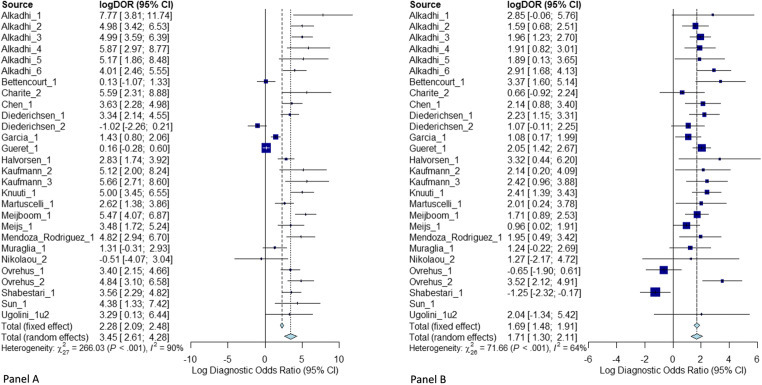
Forest plots for CTA (panel A) and Agatston score (panel B)

Frequency of patients per Agatston score subgroup of 1–100, 101–400, 401–1000, and > 1000 (Appendix Table 5) was as follows: 702, 551, 338, and 261 patients, respectively. The diagnostic accuracy of CTA was not significantly different between subgroups with Agatston scores of 1–100, 101–400, 401–1000, and > 1000 (Fig. 6) with overall diagnostic accuracies of 79.6% (95% CI: 76.7 to 82.6%), 79.9% (95% CI: 76.5 to 83.2%), 77.8 (95% CI: 73.4 to 82.2%), and 75.1% (95% CI: 69.9 to 80.3%), respectively.

## Discussion

The easy applicability, short examination time, low radiation exposure, and the fact that no contrast medium is required make the Agatston score a promising prognostic test for the evaluation of suspected CAD and prediction of cardiovascular events in clinical routine. However, the diagnostic performance of the Agatston score for detecting CAD has not been examined as intensively as that of CTA [9]. We therefore compared the diagnostic performance of the Agatston score with CTA in symptomatic patients with stable chest pain.

### Clinical implications

CTA performed better than the Agatston score in general, and diagnostic accuracy of CTA was accurate in all subanalyses, independent of the total calcium burden. Hence, CTA is superior to the Agatston score for CAD diagnosis. Moreover, we found that a zero Agatston score does not confidentially rule out obstructive CAD in symptomatic stable chest pain patients. Further, performance of CTA provides more additional information and may result in further therapeutic steps. CTA is able to show anatomy of coronary artery including diagnosis of myocardial bridging if present for example [48], but also characterization of plaques and plaque features is one of the major advantages of CTA including indication of and adherence to lipid lowering agents in patients with plaques [49]. As a result, we suggest that current international guidelines should include a consistent recommendation to always use CTA in patients with suspected CAD for ruling out obstructive CAD.

### Comparison with previous studies

Published studies provide conflicting evidence on the benefit of coronary artery calcium scoring for diagnosing CAD in clinical routine [50–57]. Controversial prevalence rates of CAD in the subgroup of zero CAC score in patients with stable chest pain can be found in the literature ranging from reported prevalence rates of 0 [52] to 20% [53]. For a better comparison of the results of our IPD meta-analysis cohort with previous studies, we performed a systematic literature search in Medline via PubMed for reported prevalence rates of CAD in patients with zero coronary artery calcium; detailed comparison of prevalence rates can be found in the Supplement (Appendix Table 6). Several studies report the CAC score to be an appropriate diagnostic test to rule out CAD in stable chest pain patients or generally symptomatic patients [50, 54] while others conclude that the Agatston score does not allow confident exclusion of obstructive CAD [55, 56]. As in our analysis, Gottlieb et al [51] found that a zero Agatston score did not reliably exclude obstructive CAD. Raff et al found lower specificity and negative predictive value in patients with severe CAC scores (> 400) in a cohort of 70 patients with suspected CAD [57]. Our comparison of diagnostic accuracy of CTA in subgroups with moderate (≤ 400) versus high (> 400) Agatston score revealed no significant difference (*p* = 0.0746, Figure 6), confirming our overall observation that CTA is superior to the Agatston score regardless of a patient’s total CAC.

To the best of our knowledge, we here for the first time investigate the diagnostic accuracy of CTA in comparison to the Agatston score and systematically analyse its performance in subgroups with increasing Agatston scores using data from an IPD meta-analysis cohort. Our approach clearly shows that the Agatston score should not be used in any case to exclude CAD. Instead, CTA should be used routinely in all symptomatic patients with suspected CAD for diagnosis of obstructive CAD independent of the amount of CAC.

### Current guidelines in light of our results

The recent European guideline for the diagnosis and management of chronic coronary syndromes by Knuuti et al [6] states that coronary artery calcium scoring may be used for cardiovascular risk assessment in asymptomatic patients (Class IIb, Level B recommendation) to improve pre-test probability calculation. In contrast, this guideline does not recommend coronary artery calcium scoring for diagnosing obstructive CAD (Level III, Class C), but recommends CTA as a first-line examination (Class I). Accordingly, a zero Agatston score has a low prevalence of obstructive CAD but is not able to exclude coronary artery stenosis caused by noncalcified stenosis. The latest version of the ACCF/AHA guideline for management of patients with stable ischemic heart disease by Finh et al [12] discusses the use of the CAC score controversially. While referencing promising evidence such as the CONFIRM registry showing 3.5% of patients with calcium score of zero having obstructive CAD [7], Finh et al very clearly conclude that more data from large study populations are needed to decide if and how CAC scoring can be used in ruling out CAD. The recent Appropriate Use Criteria introduced by Wolk et al [58] for cardiac CT propose rating the use of the Agatston score for further diagnostic decisions as rarely appropriate in symptomatic patients independent of the pre-test probability. In patients with repeated testing Agatston scores above 400, Taylor et al discourage its use for decision making. Conversely, our analysis found CTA performance not to be affected by the total amount of CAC.

Still, a consistent guideline recommendation for the use of the Agatston score in patients with a score unequal zero is missing.

In summary, the results of the COME-CCT Consortium support the current guideline of the American College of Cardiology and the European Society of Cardiology in the general message. Following, referring to the results of the COME-CCT Consortium, we call for a consistent statement and implementation for use of the CACS worldwide and cross-guidelines. With the results of our worldwide collaborative analysis, we show that it is possible to draw more specific conclusions for the use of the Agatston score and we can provide valuable scientific evidence with our IPD meta-analysis as called for by the abovementioned guidelines. Based on our results, we recommend to always perform CTA to reliably exclude CAD regardless of the total amount of coronary artery calcium measured by the Agatston score, especially in patients with a zero Agatston score. Also, in patients with an increased Agatston score, CTA might be considered. Alternatively, the Agatston score can be omitted entirely in diagnosing CAD in symptomatic patients, while keeping in mind that the Agatston score might be a useful predictor of cardiovascular events.

### Limitations

Although Agatston score analysis was a prospectively defined outcome measure of the COME-CCT Consortium, only 45% of the sites contributed data for this subanalysis, which might compromise generalizability despite the use of a well-characterized multicentre dataset. Thus, the distribution of countries and sites in this subanalysis differs from the entire study population. There was a relatively high proportion of nondiagnostic CTA examinations of 11%, which were handled in an intention-to-diagnose approach using a worst-case scenario. This may have reduced overall diagnostic performance especially when taking into account that 41% of the CT examinations were performed on CT scanners with less than 64 detector rows. Thus, accuracy of clinical CTA performed on scanner with at least 64 rows is likely higher while the accuracy of the Agatston score remains unchanged. Further technical progress in equipment and wider distribution of CTA will result in even more precision in future years, and probably decreasing the number of nondiagnostic examinations. In contrast to the current ESC guidelines (6), the proportion of patients with high pre-test probability in this analysis was relatively high with 22.7%. Inclusion criteria for the international COME-CCT Consortium were predefined before current ESC guideline; this should be highlighted in regard to the present cohort and comparability to other study cohorts. Additionally, vessel-/coronary segment-specific data, high-risk plaque features and follow-up data including occurrence of cardiovascular events were not assessed in our worldwide multicentre cohort.

### Conclusion

CTA shows an overall superior diagnostic accuracy compared with the Agatston score for the detection of CAD in symptomatic patients with stable chest pain and a clinical indication for ICA. These findings hold across patients with different Agatston scores including those without coronary artery calcium. Furthermore, the diagnostic accuracy of CTA appears to be the same in patients with moderately and highly increased Agatston scores.

## Supplementary Information

ESM 1 (DOCX 99 kb)

## Abbreviations

CAC: Coronary artery calcium
CAD: Coronary artery disease
CI: Confidence interval
COME-CCT: Collaborative Meta-Analysis of Cardiac CT
CTA: Computed tomography angiography
ICA: Invasive coronary angiography
IPD: Individual patient data
IQR: Interquartile range
PTP: Pre-test probability
SD: Standard deviation

## Declarations

### Guarantor

The scientific guarantor of this publication is Prof. Dr. Marc Dewey.

### Statistics

At least two of the authors have significant statistical knowledge.

### Methodology

- Prospective
- Diagnostic or prognostic study
- Multicentre study
