# Correction to: Computed tomography angiography versus Agatston score for diagnosis of coronary artery disease in patients with stable chest pain: individual patient data meta-analysis of the international COME-CCT Consortium

**DOI:** 10.1007/s00330-022-08760-0

**Published:** 2022-04-25

**Authors:** Viktoria Wieske, Mario Walther, Benjamin Dubourg, Hatem Alkadhi, Bjarne L. Nørgaard, Matthijs F. L. Meijs, Axel C. P. Diederichsen, Yung-Liang Wan, Hans Mickley, Konstantin Nikolaou, Abbas A. Shabestari, Bjørn A. Halvorsen, Eugenio Martuscelli, Kai Sun, Bernhard A. Herzog, Roy P. Marcus, Sebastian Leschka, Mario J. Garcia, Kristian A. Ovrehus, Juhani Knuuti, Vladymir Mendoza-Rodriguez, Nuno Bettencourt, Simone Muraglia, Ronny R. Buechel, Philipp A. Kaufmann, Elke Zimmermann, Jean-Claude Tardif, Matthew J. Budoff, Peter Schlattmann, Marc Dewey, on behalf of the COME-CCT Consortium

**Affiliations:** 1 Department of Radiology, Charité - Universitätsmedizin Berlin, Charitéplatz 1, 10117 Berlin, Germany; 2 Department of Fundamental Sciences, Jena University of Applied Sciences, Jena, Germany; 3 Cardiac Imaging Unit, Department of Radiology, Rouen University Hospital, Rouen, France; 4 Institute of Diagnostic and Interventional Radiology, University Hospital Zurich, Zurich, Switzerland; 5 Department of Cardiology, Aarhus University Hospital, Aarhus, Denmark; 6 Department of Cardiology, University Medical Centre Utrecht, Utrecht, Netherlands; 7 Department of Cardiology, Odense University Hospital, Odense, Denmark; 8 Medical Imaging and Radiological Sciences, College of Medicine, Chang Gung University, Chang Gung Memorial Hospital at Linkou, Taoyaun City, Taiwan; 9 Department of Diagnostic and Interventional Radiology, University Hospital of Tübingen, Tübingen, Germany; 10 Modarres Hospital, Shahid Beheshti University of Medical Sciences, Tehran, Iran; 11 Department of Cardiology, Ostfold Hospital Trust, Grålum, Norway; 12 Department of Internal Medicine, University of Rome Tor Vergata, Rome, Italy; 13 Department of Radiology, Baotou Central Hospital, Baotou, Inner Mongolia Province China; 14 HeartClinic Lucerne, Lucerne, Switzerland; 15 Cantonal Hospital of Lucerne, Lucerne, Switzerland; 16 Department of Radiology, Kantonsspital St Gallen, St Gallen, Switzerland; 17 Department of Cardiology, Montefiore, University Hospital for the Albert Einstein College of Medicine, New York, NY USA; 18 Turku University Hospital and University of Turku, Turku, Finland; 19 Department of Cardiology, National Institute of Cardiology and Cardiovascular Surgery, Havana, Cuba; 20 Department of Cardiology, Centro Hospitalar de Vila Nova de Gaia, Vila Nova de Gaia, Portugal; 21 Department of Cardiology, S Chiara Hospital, Trento, Italy; 22 Department of Nuclear Medicine, University Hospital Zurich, Zurich, Switzerland; 23 Montreal Heart Institute, Université de Montréal, Montréal, Canada; 24 Los Angeles Biomedical Research Institute, Torrance, CA USA; 25 Institute of Medical Statistics, Computer Sciences and Data Science, University Hospital of Friedrich Schiller University Jena, Jena, Germany

**Correction to: European Radiology**

10.1007/s00330-022-08619-4

The original version of this article, published on 10 March 2022, unfortunately contained a mistake. The author name Bjarne L. Nørgaard was incorrectly given as Bjarne L. Nørgard. The original article has been corrected.

