# Supplementary material for: Computed tomography angiography versus Agatston score for diagnosis of coronary artery disease in patients with stable chest pain: individual patient data meta-analysis of the international COME-CCT Consortium
Source: Eur Radiol. 2022 Mar 10;32(8):5233–45. doi: 10.1007/s00330-022-08619-4 (PMC9279219; doi:10.1007/s00330-022-08619-4)
Supplement: Supplementary file 1 — (DOCX 99 kb) [file 330_2022_8619_MOESM1_ESM.docx]

**SUPPLEMENTARY MATERIALS**

COME-CCT Members

| **Site** | **Names** |
| --- | --- |
|  |  |
| Charité Universitätsmedizin Berlin,  Berlin, Germany | Marc Dewey Robert Haase  Michael Laule Matthias Rief  Robert Röhle  Georg Schuetz  Viktoria Wieske Benjamin Weickert  Simon Andrzejewski  Elke Zimmermann |
|  |  |
| Jena University of Applied Sciences, Jena, Germany | Mario Walther |
|  |  |
| Centro Cardiologico Monzino IRCCS (D.A., G.P.), University of Milan (D.A.), Milan, Italy | Daniele Andreini Gianluca Pontone |
|  |  |
| Henri Mondor Hospital, University Paris Est Créteil, Créteil, France | Pascal Gueret |
|  |  |
| University Hospital Zurich, Zurich, Switzerland | Hatem Alkadhi Ronny Ralf Buechel Bernhard Herzog Philipp Kaufmann |
|  |  |
| Ludwig-Maximilians-Universität München,  Munich, Germany | Jörg Hausleiter |
|  |  |
| Kantonsspital St. Gallen,  St. Gallen, Switzerland | Sebastian Leschka |
|  |  |
| Montefiore, the University Hospital for the Albert Einstein College of Medicine,  New York, USA | Mario Garcia |
|  |  |
| Clinique Universitaires St. Luc, Institut de Recherche Clinique et Expérimentale,  Brussels, Belgium | Bernhard Gerber |
|  |  |
| Medical University of South Carolina,  Charleston, USA | U. Joseph Schoepf |
|  |  |
| Modarres Hospital, Shahid Beheshti University of Medical Sciences,  Tehran, Iran | Abbas Arjmand Shabestari |
|  |  |
| Aarhus Universtity Hostipat,  Aarhus, Denmark | Bjarne Nørgaard |
|  |  |
| Turku University Hospital and University of Turku,  Turku, Finland | Juhani Knuuti |
|  |  |
| Odense University Hospital,  Odense, Denmark | Kristian Altern Ovrehus  Axel Cosmus Pyndt Diederichsen  Hans Mickley |
|  |  |
| Faculty of Medicine, University of Tsukuba,  Tsukuba, Japan | Akira Sato |
|  |  |
| Rabin Medical Center, Sackler Faculty of Medicine, Tel-Aviv University,  Tel-Aviv, Israel | Ashraf Hamdan |
|  |  |
| National Institute of Cardiology and Cardiovascular Surgery,  Havana, Cuba | Vladimir Mendoza-Rodriguez |
|  |  |
| University of São Paulo,  São Paulo, Brazil | Carlos Rochitte |
|  |  |
| Ostfold Hospital Trust,  Grålum, Norway | Bjørn Arild Halvorsen |
|  |  |
| College of Medicine, Chang Gung University, Chang Gung Memorial Hospital at Linkou,  Taoyaun City, Taiwan | Yung Liang Wan |
|  |  |
| Universitätsklinikum Giessen und Marburg GmbH,  Giessen, Germany | Johannes Rixe |
|  |  |
| Glasgow Royal Infirmary and Stobhill Hospital,  Glasgow, United Kingdom | Shona M. Jenkins |
|  |  |
| University of Rome Tor Vergata,  Rome, Italy | Eugenio Martuscelli |
|  |  |
| Beijing Anzhen Hospital,  Beijing, China | Zhaqoi Zhang Lin Yang |
|  |  |
| Main-Taunus-Kliniken,  Bad Soden, Germany | Christoph Langer |
|  |  |
| University Hospital of Tübingen,  Tübingen, Germany | Konstantin Nikolaou Roy Paul Marcus |
|  |  |
| Lady Davis Carmel Medical Center,  Haifa, Israel | David A. Halon |
|  |  |
| Natioanl Heart and Blood Institue, National Institutes of Health, Bethesda, USA | Marcus Y. Chen |
|  |  |
| Centro Hospitalar de Vila Nova de Gaia/Espinho,  Gaia, Portugal | Nuno Bettencourt |
|  |  |
| St. Luke's International Hospital,  Tokyo, Japan | Hiroyuki Niinuma |
|  |  |
| Baotou Central Hospital, Inner Mongolia Province , China | Kai Sun |
|  |  |
| University Hospital Pitié-Salpêtrière,  Paris, France | Réda Jakamy |
|  |  |
| University of Ottawa (B.C., F.R.), Heart Institute (B.C.),  Ottawa, Canada | Benjamin Chow Frank Rybicki |
|  |  |
| S. Chiara Hospital,  Trento, Italy | Simone Muraglia |
|  |  |
| Université de Montréal,  Montréal, Canada | Jean-Claude Tardif |
|  |  |
| Albert Einstein Hospital,  São Paulo, Brazil | Cesar Nomura |
|  |  |
| The Heart Center, Rigshospitalet, University of Copenhagen,  Copenhagen, Denmark | Klaus F. Kofoed |
|  |  |
| The Johns Hopkins Hospital, Johns Hopkins University, Baltimore, USA | Armin A. Zadeh |
|  |  |
| Mie University Hospital,  Tsu, Japan | Kakuya Kitagawa |
|  |  |
| Beth Israel Deaconess Medical Center, Harvard University,  Boston, USA | Roger Laham |
|  |  |
| Keio University,  Tokyo, Japan | Masahiro Jinzaki |
|  |  |
| Mount Elizabeth Hospital,  Singapore, Singapore | John Hoe |
|  |  |
| Leiden University Medical Center,  Leiden, The Netherlands | Arthur Scholte |
|  |  |
| University Health Network, University of Toronto,  Toronto, Canada | Narinder Paul |
|  |  |
| Iwate Medical University,  Morioka, Japan | Kunihiro Yoshioka |
|  |  |
| National Heart Center,  Singapore, Singapore | Swee Yaw Tan |
|  |  |
| Catholic University of Paraná,  Curitiba, Brazil | Tiago Augusto Magalhães |
| University Hospital of Friedrich Schiller University Jena, Jena, Germany | Peter Schlattmann |

**Data Management Team**

Robert Röhle, Mario Walther, Robert Haase, Georg M. Schuetz, Viktoria Wieske, and Marc Dewey.

**Steering Committee**

The steering committee consists of four clinical experts for computed tomography: Stephan Achenbach, Erlangen, Germany; Matthew Budoff, Los Angeles, California, USA; Mario J Garcia, New York, New York, USA; Marc Dewey, Berlin, Germany, and one clinical expert for invasive coronary angiography: Michael Laule, Berlin, Germany. The steering committee is completed by the project’s statistician Peter Schlattmann, Jena, Germany.

**Coordinating Center**

Georg M. Schuetz, Robert Röhle, Robert Haase, Michael Laule, Viktoria Wieske, Marc Dewey (principal investigator).

**REFERENCES OF INCLUDED STUDIES**

1. Alkadhi H, Scheffel H, Desbiolles L et al. Dual-source computed tomography coronary angiography: influence of obesity, calcium load, and heart rate on diagnostic accuracy. European heart journal 2008;29:766-76.

2. Alkadhi H, Stolzmann P, Desbiolles L et al. Low-dose, 128-slice, dual-source CT coronary angiography: accuracy and radiation dose of the high-pitch and the step-and-shoot mode. Heart (British Cardiac Society) 2010;96:933-8.

3. Bettencourt N, Rocha J, Carvalho M et al. Multislice computed tomography in the exclusion of coronary artery disease in patients with presurgical valve disease. Circulation Cardiovascular imaging 2009;2:306-13.

4. Bonmassari R, Muraglia S, Centonze M, Coser D, Stoppa G, Disertori M. Noninvasive detection of coronary artery stenosis with 16-slice spiral computed tomography in a population at low to moderate risk for coronary artery disease. Journal of cardiovascular medicine (Hagerstown, Md) 2006;7:817-25.

5. Chen CC, Chen CC, Hsieh IC et al. The effect of calcium score on the diagnostic accuracy of coronary computed tomography angiography. The international journal of cardiovascular imaging 2011;27 Suppl 1:37-42.

6. Dewey M, Zimmermann E, Deissenrieder F et al. Noninvasive coronary angiography by 320-row computed tomography with lower radiation exposure and maintained diagnostic accuracy: comparison of results with cardiac catheterization in a head-to-head pilot investigation. Circulation 2009;120:867-75.

7. Diederichsen AC, Petersen H, Jensen LO et al. Diagnostic value of cardiac 64-slice computed tomography: importance of coronary calcium. Scandinavian cardiovascular journal : SCJ 2009;43:337-44.

8. Garcia MJ, Lessick J, Hoffmann MH. Accuracy of 16-row multidetector computed tomography for the assessment of coronary artery stenosis. Jama 2006;296:403-11.

9. Gueret P, Deux JF, Bonello L et al. Diagnostic performance of computed tomography coronary angiography (from the Prospective National Multicenter Multivendor EVASCAN Study). The American journal of cardiology 2013;111:471-8.

10. Halvorsen BA, Rodevand O, Hagen G, Herud E, Mielczarek W, Molstad P. [Angiography with 64-channel CT upon suspicion of stable coronary disease]. Tidsskr Nor Laegeforen 2008;128:2172-6.

11. Herzog BA, Wyss CA, Husmann L et al. First head-to-head comparison of effective radiation dose from low-dose 64-slice CT with prospective ECG-triggering versus invasive coronary angiography. Heart (British Cardiac Society) 2009;95:1656-61.

12. Husmann L, Herzog BA, Burger IA et al. Usefulness of additional coronary calcium scoring in low-dose CT coronary angiography with prospective ECG-triggering impact on total effective radiation dose and diagnostic accuracy. Academic radiology 2010;17:201-6.

13. Kajander S, Joutsiniemi E, Saraste M et al. Cardiac positron emission tomography/computed tomography imaging accurately detects anatomically and functionally significant coronary artery disease. Circulation 2010;122:603-13.

14. Leschka S, Alkadhi H, Plass A et al. Accuracy of MSCT coronary angiography with 64-slice technology: first experience. European heart journal 2005;26:1482-7.

15. Leschka S, Scheffel H, Desbiolles L et al. Combining dual-source computed tomography coronary angiography and calcium scoring: added value for the assessment of coronary artery disease. Heart (British Cardiac Society) 2008;94:1154-61.

16. Leschka S, Scheffel H, Husmann L et al. Effect of decrease in heart rate variability on the diagnostic accuracy of 64-MDCT coronary angiography. AJR American journal of roentgenology 2008;190:1583-90.

17. Martuscelli E, Romagnoli A, D'Eliseo A et al. Accuracy of thin-slice computed tomography in the detection of coronary stenoses. European heart journal 2004;25:1043-8.

18. Meijboom WB, Meijs MF, Schuijf JD et al. Diagnostic accuracy of 64-slice computed tomography coronary angiography: a prospective, multicenter, multivendor study. Journal of the American College of Cardiology 2008;52:2135-44.

19. Meijboom WB, Mollet NR, Van Mieghem CA et al. Pre-operative computed tomography coronary angiography to detect significant coronary artery disease in patients referred for cardiac valve surgery. Journal of the American College of Cardiology 2006;48:1658-65.

20. Meijboom WB, Weustink AC, Pugliese F et al. Comparison of diagnostic accuracy of 64-slice computed tomography coronary angiography in women versus men with angina pectoris. The American journal of cardiology 2007;100:1532-7.

21. Mendoza-Rodriguez V, Llerena LR, Llerena LD et al. Ischemic heart disease diagnosed by 64 slice computed tomography coronary angiography.

22. Ovrehus KA, Jensen JK, Mickley HF et al. Comparison of usefulness of exercise testing versus coronary computed tomographic angiography for evaluation of patients suspected of having coronary artery disease. The American journal of cardiology 2010;105:773-9.

23. Ovrehus KA, Munkholm H, Bottcher M, Botker HE, Norgaard BL. Coronary computed tomographic angiography in patients suspected of coronary artery disease: impact of observer experience on diagnostic performance and interobserver reproducibility. Journal of cardiovascular computed tomography 2010;4:186-94.

24. Scheffel H, Alkadhi H, Plass A et al. Accuracy of dual-source CT coronary angiography: First experience in a high pre-test probability population without heart rate control. European radiology 2006;16:2739-47.

25. Shabestari AA, Abdi S, Akhlaghpoor S et al. Diagnostic performance of 64-channel multislice computed tomography in assessment of significant coronary artery disease in symptomatic subjects. The American journal of cardiology 2007;99:1656-61.

26. Sun K, Han RJ, Cui LF et al. Feasibility and Diagnostic Accuracy for Assessment of Coronary Artery Stenosis of Prospectively Electrocardiogram-gated High-pitch Spiral Acquisition Mode Dual-source CT Coronary Angiography in Patients with Relatively Higher Heart Rates: in Comparison with Catheter Coronary Angiography. Chinese medical sciences journal = Chung-kuo i hsueh k'o hsueh tsa chih / Chinese Academy of Medical Sciences 2013;27:213-9.

27. Ugolini P, Pressacco J, Lesperance J et al. Evaluation of coronary atheroma by 64-slice multidetector computed tomography: Comparison with intravascular ultrasound and angiography. The Canadian journal of cardiology 2009;25:641-7.*

28. Unpublished study #1, Diederichsen AC.

29. Unpublished study #2, Ugolini P.*

*Studies were combined to one dataset due to small sample size of the available patients in each original dataset.

**APPENDIX RESULTS TABLES**

**Appendix Table 1. Direct Comparison of CTA excluding NDX and CTA including NDX versus the Reference Standard of Invasive Coronary Angiography**

|  | **Invasive Coronary Angiography** | |
| --- | --- | --- |
|  | Positive | Negative |
| **CTA excluding NDX** |  |  |
| Positive | 943 (95.0%) | 185 (15.6%) |
| Negative | 50 (5.0%) | 999 (84.4%) |
| Total | 993 (100.0%) | 1,184 (100.0%) |
| **CTA including NDX*** |  |  |
| Positive | 943 (85.7%) | 353 (26.1%) |
| Negative | 157 (14.3%) | 999 (73.9%) |
| Total | 1,100 (100.0%) | 1,352 (100.0%) |

Values are numbers (percentages) unless stated otherwise.

* With nondiagnostic CT (n=275) defined as positive. NDX – nondiagnostic CT.

**Appendix Table 2. Direct Comparison of Agatston score excluding NDX and Agatston score including NDX versus the Reference Standard of Invasive Coronary Angiography**

|  | **Invasive Coronary Angiography** | |
| --- | --- | --- |
|  | Positive | Negative |
| **Agatston Score excluding NDX** |  |  |
| Positive | 387 (39.0%) | 124 (10.5%) |
| Negative | 606 (61.0%) | 1060 (89.5%) |
| Total | 993 (100.0%) | 1,184 (100.0%) |
| **Agatston Score including NDX*** |  |  |
| Positive | 443 (40.3%) | 156 (11.5%) |
| Negative | 657 (59.7%) | 1,196 (88.5%) |
| Total | 1,100 (100.0%) | 1,352 (100.0%) |

Values are numbers (percentages) unless stated otherwise.

* With nondiagnostic CT (n=275) defined as positive. NDX – nondiagnostic CT.

**Appendix Table 3. Diagnostic Performance of CTA and Agatston Score excluding NDX* on the Patient Level**

|  | **CTA**  *n/total n (%[95% CI*])* | **Agatston Score**  *n/total n (%[95% CI*])* | **p-value*** |
| --- | --- | --- | --- |
| Diagnostic accuracy | 1,942/2,177 (90.0% [87.6-92.0]) | 1,447/2,177 (67.3% [62.3-71.8]) | <0.0001 |
| Sensitivity | 943 / 993 (95.4% [93.7-96.7]) | 387/993 (39.6% [34.8-44.5]) | <0.0001 |
| Specificity | 999 / 1,184 (84.3% [81.2-87.1]) | 1,060/1,184 (89.5% [87.0-91.6]) | <0.0001 |
| Negative predictive value | 999 / 1,049 (95.0% [92.7-96.6]) | 1,060/1,666 (63.4% [56.0-70.2]) | <0.0001 |
| Positive predictive value | 943 / 1,128 (84.2% [79.2-88.1]) | 387/511 (77.1% [70.2-82.7]) | 0.0007 |
| Positive likelihood ratio | 5.94 [4.26 – 8.28] | 3.21 [2.38 – 4.32] |  |
| Negative likelihood ratio | 0.08 [0.06 – 0.11] | 0.69 [0.61 – 0.77] |  |
| DOR | 100.26 [57.65 – 174.35] | 5.31 [3.45 – 8.16] |  |

* CI - confidence interval. Estimates, 95% CI, and p-values are based on a model with study-specific random intercept taking test correlation within patients into account and were determined as described for unclustered data.

Positive likelihood ratio, negative likelihood ratio and diagnostic odds ratio (DOR) based on random effect models for each test.

**Appendix Table 4. Distribution of angina pectoris type by Agatston score subgroup.**

|  | **Typical angina** | | | | **Atypical angina** | | | | **Nonanginal chest pain** | | | | **Other chest discomfort** | | | | **Independent of Symptoms** | | | |
| --- | --- | --- | --- | --- | --- | --- | --- | --- | --- | --- | --- | --- | --- | --- | --- | --- | --- | --- | --- | --- |
|  | **CAD** | | **No CAD** | | **CAD** | | **No CAD** | | **CAD** | | **No CAD** | | **CAD** | | **No CAD** | | **CAD** | | **No CAD** | |
|  | **N** | **%** | **N** | **%** | **N** | **%** | **N** | **%** | **N** | **%** | **N** | **%** | **N** | **%** | **N** | **%** | **N** | **%** | **N** | **%** |
| **All Patients** | 588 | 54.5 | 490 | 45.5 | 252 | 33.8 | 494 | 66.2 | 183 | 39.2 | 284 | 60.8 | 77 | 47.8 | 84 | 52.2 | 1,100 | 44.9 | 1352 | 55.1 |
| **Ag. Score Zero** | 47 | 21.6 | 171 | 78.4 | 17 | 8.2 | 190 | 91.8 | 27 | 19.9 | 109 | 80.1 | 10 | 25.6 | 29 | 74.4 | 101 | 16.8 | 499 | 83.2 |
| **Ag. Score <100** | 118 | 40.7 | 172 | 59.3 | 50 | 22.9 | 168 | 77.1 | 62 | 42.8 | 83 | 57.2 | 20 | 40.8 | 29 | 59.2 | 250 | 35.6 | 452 | 64.4 |
| **Ag. Score 100 to 400** | 157 | 63.1 | 92 | 36.9 | 77 | 45.0 | 94 | 55.0 | 48 | 51.6 | 45 | 48.4 | 24 | 63.2 | 14 | 36.8 | 306 | 55.5 | 245 | 44.5 |
| **Ag. Score 401 to 1000** | 143 | 80.3 | 35 | 19.7 | 58 | 69.9 | 25 | 30.1 | 26 | 44.8 | 32 | 55.2 | 11 | 57.9 | 8 | 42.1 | 238 | 70.4 | 100 | 29.6 |
| **Ag. Score >1000** | 123 | 86.0 | 20 | 14.0 | 50 | 74.6 | 17 | 25.4 | 20 | 57.1 | 15 | 42.9 | 12 | 75.0 | 4 | 25.0 | 205 | 78.5 | 56 | 21.5 |
|  |  |  |  |  |  |  |  |  |  |  |  |  |  |  |  |  |  |  |  |  |
| **Ag. Score ≤400** | 322 | 42.5 | 435 | 57.5 | 144 | 24.2 | 452 | 75.8 | 137 | 36.6 | 237 | 63.4 | 54 | 42.9 | 72 | 57.1 | 657 | 35.5 | 1196 | 64.5 |
| **Ag. Score >400** | 266 | 82.9 | 55 | 17.1 | 108 | 72.0 | 42 | 28.0 | 46 | 49.5 | 47 | 50.5 | 23 | 65.7 | 12 | 34.3 | 443 | 74.0 | 156 | 26.0 |

Ag. Score – Agatston score; CAD – coronary artery disease.

**Appendix Table 5. Characteristics of the 2452 Patients* by Agatston Score Subgroup.**

| **Characteristic** | **Agatston Score Subgroup** | | | | | | |
| --- | --- | --- | --- | --- | --- | --- | --- |
|  | **Zero** | **1 to <100** | **100 to 400** | **401 to 1000** | **>1000** | **≤400** | **>400** |
| Patients (N) per Subgroup | 600 | 702 | 551 | 338 | 261 | 1853 | 599 |
| **Age in years, n (SD)** | 57 ± 11 | 60 ± 10 | 63 ± 9 | 65 ± 9 | 67 ± 9 | 60 ±10 | 66 ± 9 |
| N | 600 | 702 | 551 | 338 | 261 | 1853 | 599 |
| Missings | 0 | 0 | 0 | 0 | 0 | 0 | 0 |
| **Male sex, n (%)** | 318 (53.0) | 454 (64.7) | 380 (69.0) | 250 (74.0) | 201 (77.0) | 1152 (62.2) | 451 (75.3) |
| N | 600 | 702 | 551 | 338 | 261 | 1853 | 599 |
| Missings | 0 | 0 | 0 | 0 | 0 | 0 | 0 |
| **Agatston Score** |  |  |  |  |  |  |  |
| Median | 0 | 25 | 219 | 607 | 1583 | 23 | 872 |
| Minimum | 0 | 1 | 100 | 401 | 1012 | 0 | 401 |
| Maximum | 0,9 | 99 | 400 | 1000 | 6210 | 400 | 6210 |
| 1st Quartile | 0 | 10 | 147 | 492 | 1264 | 0 | 576 |
| 3rd Quartile | 0 | 50 | 293 | 756 | 2400 | 127 | 1434 |
| N | 600 | 702 | 551 | 338 | 261 | 1853 | 599 |
| Missings | 0 | 0 | 0 | 0 | 0 | 0 | 0 |
| **Hypertension, n (%)** | 252 (43.0) | 362 (54.0) | 339 (63.7) | 205 (61.6) | 174 (66.9) | 953 (53.3) | 379 (63.9) |
| N | 586 | 670 | 532 | 333 | 260 | 1788 | 593 |
| Missings | 14 | 32 | 19 | 5 | 1 | 65 | 6 |
| **Diabetes, n (%)** | 71 (12.1) | 108 (15.9) | 117 (21.9) | 76 (22.8) | 70 (26.8) | 296 (16.4) | 146 (24.5) |
| N | 586 | 680 | 534 | 334 | 261 | 1800 | 595 |
| Missings | 14 | 22 | 17 | 4 | 0 | 53 | 4 |
| **Hyperlipidemia, n (%)** | 221 (41.2) | 337 (52.1) | 297 (57.1) | 185 (57.6) | 132 (53.0) | 855 (50.2) | 317 (55.6) |
| N | 536 | 647 | 520 | 321 | 249 | 1703 | 570 |
| Missings | 64 | 55 | 31 | 17 | 12 | 150 | 29 |

| **Characteristic** | **Agatston Score Subgroup** | | | | | | |
| --- | --- | --- | --- | --- | --- | --- | --- |
|  | **Zero** | **1 to <100** | **100 to 400** | **400 to 1000** | **>1000** | **≤400** | **>400** |
| **Current Smoker, n (%)** | 160 (27.3) | 203 (29.9) | 170 (31.8) | 97 (29.0) | 78 (29.9) | 533 (29.6) | 175 (29.4) |
| N | 587 | 680 | 534 | 334 | 261 | 1801 | 595 |
| Missings | 13 | 22 | 17 | 4 | 0 | 52 | 4 |
| **Body Mass Index**^†^**, n (SD)** | 26.2 ± 4.1 | 26.9 ± 4.4 | 26.8 ± 4.0 | 27.2 ± 4.0 | 27.2 ± 4.0 | 26.6 ± 4.2 | 27.2 ± 4.0 |
| N | 592 | 698 | 542 | 335 | 258 | 1832 | 593 |
| Missings | 8 | 4 | 9 | 3 | 3 | 21 | 6 |
| **Prevalence of  obstructive CAD, n (%)** | 101 (16.8) | 250 (35.6) | 306 (55.5) | 238 (70.4) | 205 (78.5) | 657 (35.5) | 443 (74.0) |
| N | 600 | 702 | 551 | 338 | 261 | 1853 | 599 |
| Missings | 0 | 0 | 0 | 0 | 0 | 0 | 0 |
| **Pretest probability, n (SD)** | 42 ± 16 | 47 ± 16 | 51 ± 16 | 55 ± 16 | 58 ± 16 | 47 ± 16 | 56 ± 16 |
| N | 600 | 702 | 551 | 338 | 261 | 1853 | 599 |
| Missings | 0 | 0 | 0 | 0 | 0 | 0 | 0 |
| **Angina pectoris classification, n (%)** |  |  |  |  |  |  |  |
| **Typical angina** | 218 (36.3) | 290 (41.3) | 249 (45.2) | 178 (52.7) | 143 (54.8) | 757 (40.9) | 321 (53.6) |
| **Atypical angina** | 207 (34.5) | 218 (31.1) | 171 (31.0) | 83 (24.6) | 67 (25.7) | 596 (32.2) | 150 (25.0) |
| **Nonanginal chest pain** | 136 (22.7) | 145 (20.7) | 93 (16.9) | 58 (17.2) | 35 (13.4) | 374 (20.2) | 93 (15.5) |
| **Other chest discomfort** | 39 (6.5) | 49 (7.0) | 38 (6.9) | 19 (5.6) | 16 (6.1) | 126 (6.8) | 35 (5.8) |
| N | 600 | 702 | 551 | 338 | 261 | 1853 | 599 |
| Missings | 0 | 0 | 0 | 0 | 0 | 0 | 0 |

* Plus-minus values are means ± SD unless otherwise stated. Percentages are based on total number of available patients (N) in the respective category. † Calculated as the weight in kilograms divided by the square of the height in meters.

**Appendix Table 6. Comparison of CAD prevalence in patients with zero CACS in different studies – A systematic literature search in Medline via PubMed***

| **Study** | **Year** | **Patient cohort** | **Total number of patients (total number for CACS analysis if different from total number)** | **Number of Patients with Zero CACS** | **Definition of obstructive CAD / (Reference standard)** | **CAD prevalence in whole study cohort** | **Reported prevalence in % of CAD in patients with CACS = 0** |
| --- | --- | --- | --- | --- | --- | --- | --- |
| Akram et al. (1) | 2009 | Retrospective analysis of patients with suspected CAD undergoing CTA and CAC scoring. 64% of the patients presented with symptoms. | 210 | 1. 70 (all) 2. subgroup symptomatic patients: 49 | >70% (ICA) | 18% | 1. 5.7% 2. 8.16% |
| AlJaroudi et al. (2) | 2019 | 1. Patients with non-specific or atypical symptoms or for routine check-up and results of stress echocardiography and CAC scoring from the executive screening program were restropectively included. Patients with normal stress echocardiography and CAC score of zero were analyzed for presence of CAD.  2. Validation cohort with 111 patients. | 1.173 2.111 | 1. 128 2. 53 | ≥70% stenosis in a major epicardial vessel or ≥ 50% stenosis in LM | 1. 12.1% 2. 1.8% | 1.0% 2.0% |
| Alqarqaz et al. (3) | 2011 | 333 symptomatic patients with zero CAC and suspected CAD, main aim: to assess the prevalence of non-calcified plaques (NCP) | 333 | 333  (55 with NCP) | >50% | NA | 1.8% |

| **Study** | **Year** | **Patient cohort** | **Total number of patients (total number for CACS analysis if different from total number)** | **Number of Patients with Zero CACS** | **Definition of obstructive CAD / (Reference standard)** | **CAD prevalence in whole study cohort** | **Reported prevalence in % of CAD in patients with CACS = 0** |
| --- | --- | --- | --- | --- | --- | --- | --- |
| Arbab-Zadeh et al. (4) | 2012 | 371 symptomatic patients from the CORE-64 study (secondary analysis including patients with CAC scores ≥600 initially excluded) for detection of obstructive CAD, known CAD in 39% | 371 | 72 | ≥50% (ICA, QCA) | 63% | 19.40% |
| Becker et al. (5) | 2007 | Symptomatic patients (typical/atypical chest pain, exertional dyspnea, unexplained heart failure) | 1347 | 259 | ≥50% (ICA) | 53% | 0.60% |
| Bom et al. (6) | 2016 | 1551 symptomatic outpatients with lo or intermediate pretest probability and suspected CAD were followed for MACE for a median of 637 days. | 1551 | 739 | >50% (CTA) | 44% | 3.10% |
| Dedic et al. (7) | 2013 | Patients with stable chest pain in a chest pain clinic planned to undergo exercise ECG, CAC scoring, CTA. Clinically driven indication and referral for ICA (n=180 patients). | 791 (756) | 281 | ≥50% (CTA or ICA) | 27% | 3.55% |
| Ergün et al. (8) | 2010 | Patients with CAC score of zero for assessment of presence and extent of plaques undergoing CTA for suspected CAD. 36% of the patients were asymptomatic. | 883 | 883 | ≥50% | NA† | 4.90% |
| **Study** | **Year** | **Patient cohort** | **Total number of patients (total number for CACS analysis if different from total number)** | **Number of Patients with Zero CACS** | **Definition of obstructive CAD / (Reference standard)** | **CAD prevalence in whole study cohort** | **Reported prevalence in % of CAD in patients with CACS = 0** |
| Gabriel et al. (9) | 2018 | Patients with CAC score of zero for evaluation of frequency of plaque burden and degree of obstruction. 40% with atypical chest pain, 25% with typical chest pain. | 367 | 367 | >50% | NA† | 4.40% |
| Gotttlieb et al. (10) | 2010 | Symptomatic patients with suspected CAD from the CORE-64 study, in the primary analysis only patients with CAC score of ≤600 were included. | 291 | 72 | ≥50% (ICA) | 56% | 19% |
| Haberl et al. (11) | 2001 | Patients with stable chest pain, suspected CAD and a referral for ICA. | 1.764 | 249 | ≥50% (ICA) | 53.0% | 2.0% |
| Hulten et al. (12) | 2014 | Retrospective cohort studies including symptomatic patients without prior CAD who underwent CAC score and CTA. Evaluation of diagnostic accuracy of CAC and CTA were performed as well as predictive value of both, CAC and CTA, for major adverse cardiovascular events. | 1.145 | 483 | 1. ≥50% (ICA) 2. ≥70% (ICA) | 25% | 1. 1.45% 2. 0.45% |

| **Study** | **Year** | **Patient cohort** | **Total number of patients (total number for CACS analysis if different from total number)** | **Number of Patients with Zero CACS** | **Definition of obstructive CAD / (Reference standard)** | **CAD prevalence in whole study cohort** | **Reported prevalence in % of CAD in patients with CACS = 0** |
| --- | --- | --- | --- | --- | --- | --- | --- |
| Kelly et al. (13) | 2008 | Retrospective review of patients undergoing CTA and CAC sore for suspected CAD for evaluation of prevalence and severity of CAD in patients with CAC score of zero. Atypical chest pain was found in 28% of the cohort. | 729 | 385 | >50% | NA† | 1.50% |
| Kim et al. (14) | 2012 | 2088 symptomatic patients who underwent CTA based on suspected CAD. | 2.088 | 1.114 | ≥50% | 21% | 4.30% |
| Liu et al. (15) | 2013 | 100 symptomatic patients to evaluate clinical significance of CAC in predicting CAD and cardiac events. Prevalence of cardiac events rate: 13% | 100 | 15 | ≥50% (ICA) | 72% | 20% |
| Meyer at al. (16) | 2012 | 383 symptomatic patients with an intermediate risk score undergoing CAC score and CTA referred by chest pain unit for suspected CAD. | 383 | 121 | ≥50% | 30% | 0% |
| Mittal et al. (17) | 2017 | 3914 patients with stable chest pain referred for CAC score or CAC score + CTA, all patients with information on CAC, of those 2730 patients with CTA. | 3.914 (2.730 with CAC+CTA) | 1.426 | ≥50% | NA | 1.7% |

| **Study** | **Year** | **Patient cohort** | **Total number of patients (total number for CACS analysis if different from total number)** | **Number of Patients with Zero CACS** | **Definition of obstructive CAD / (Reference standard)** | **CAD prevalence in whole study cohort** | **Reported prevalence in % of CAD in patients with CACS = 0** |
| --- | --- | --- | --- | --- | --- | --- | --- |
| Moradi et al. (18) | 2015 | Retrospective study with 2000 patients undergoing CTA clinically referred for ruling out CAD, patients with zero CAC score were included. | 2000 | 385 | >50% | NA† | 1,60% |
| Nieman et al. (19) | 2009 | Patients with stable chest pain and no history of CAD scheduled for CTA based on suspected CAD. | 471 (463) | 175 (total, 5 non-diagnostic) | ≥50% (ICA, QCA) | 58% | 1,77% |
| Parsons et al. (20) | 2017 | Two year retrospective cohort study of UK military patients undergoing CAC score and CTA for suspected CAD. | 44 | 21 | ≥50% (CTA) | 30% | 14,20% |
| Schubaeck et al. (21) | 2016 | Retrospective review of 2614 with suspected CAD (40% without symptoms, unknown symptoms in 7%) and performed CAC score + CTA. | 2614 | 1032 | ≥75% (ICA) | 15,30% | 2,60% |
| Sosnowski et al. (22) | 2009 | Retrospective evaluation of symptomatic patients with CAC of zero, intermediate risk of CAD and suspected CAD undergoing CTA. | 166 | 166 | >50% (CTA) | NA† | 2% |
| Ueda et al. (23) | 2012 | Patients with chest pain and or signs of cardiac disease undergoing CTA for suspected CAD. | 753 | 260 | >50% | 25,10% | 3,10% |

*References of the original studies listed here can be found below under References Appendix Table 6. Studies with available absolute and/or relative numbers of the prevalence of obstructive CAD in stable chest pain patients with zero CACS and suspected CAD were included in this table.

NA† - Either only patients with CAC zero included or only patients with CAC zero analysed; Abbreviations: CAD – Coronary artery disease; CACS – coronary artery calcium score; CAC – coronary artery calcium; LM – Left main; only >/>= 50% presented if subgroup analysis e.g. for >50% and >70% available.

**APPENDIX RESULTS FIGURES**

**Appendix Figure 1A. Funnel plots for CTA (with 95% confidence interval)**

**
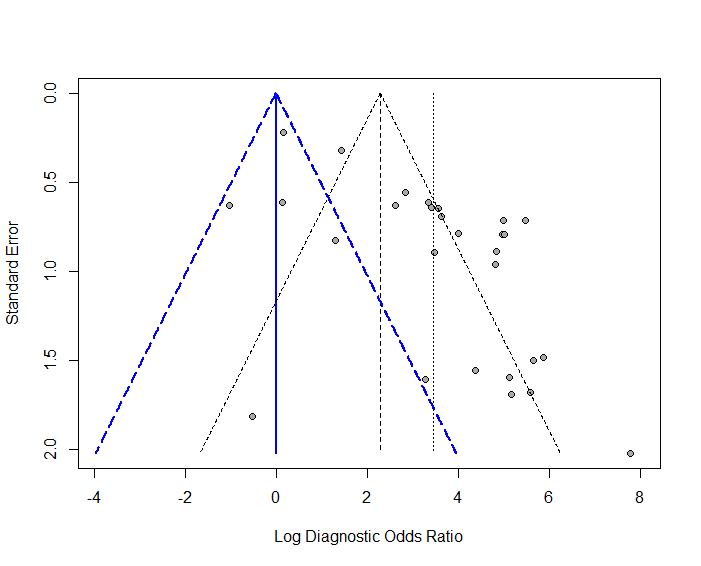
**

Deeks test was performed to exclude potential publication bias showing no significant difference between the included studies (p = 0.1495), indicating no potential publication bias.

**Appendix Figure 1B. Funnel plots for Agatston Score (with 95% confidence interval)**

**
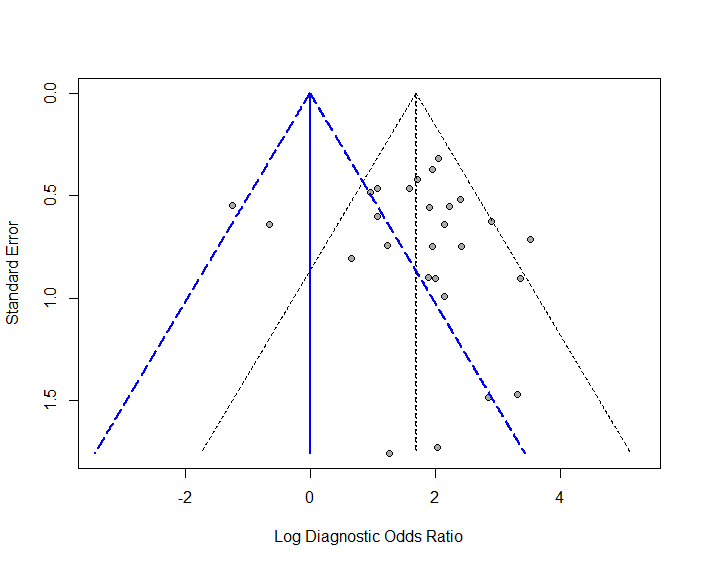
**

Deeks test was performed to exclude potential publication bias showing no significant difference between the included studies (p = 0.9728), indicating no potential publication bias.

**References Appendix Table 6**

1. Akram K, O'Donnell RE, King S, Superko HR, Agatston A, Voros S. Influence of symptomatic status on the prevalence of obstructive coronary artery disease in patients with zero calcium score. Atherosclerosis. 2009;203(2):533-7. 10.1016/j.atherosclerosis.2008.07.008

2. AlJaroudi W, Mansour MJ, Chedid M, Hamoui O, Asmar J, Mansour L, et al. Incremental value of stress echocardiography and computed tomography coronary calcium scoring for the diagnosis of coronary artery disease. Int J Cardiovasc Imaging. 2019;35(6):1133-9. 10.1007/s10554-019-01577-x

3. Alqarqaz M, Zaidan M, Al-Mallah MH. Prevalence and predictors of atherosclerosis in symptomatic patients with zero calcium score. Academic radiology. 2011;18(11):1437-41. 10.1016/j.acra.2011.07.012

4. Arbab-Zadeh A, Miller JM, Rochitte CE, Dewey M, Niinuma H, Gottlieb I, et al. Diagnostic accuracy of computed tomography coronary angiography according to pre-test probability of coronary artery disease and severity of coronary arterial calcification. The CORE-64 (Coronary Artery Evaluation Using 64-Row Multidetector Computed Tomography Angiography) International Multicenter Study. Journal of the American College of Cardiology. 2012;59(4):379-87. 10.1016/j.jacc.2011.06.079

5. Becker A, Leber A, White CW, Becker C, Reiser MF, Knez A. Multislice computed tomography for determination of coronary artery disease in a symptomatic patient population. Int J Cardiovasc Imaging. 2007;23(3):361-7. 10.1007/s10554-006-9189-1

6. Bom MJ, Van der Zee PM, Van der Zant FM, Knol RJ, Cornel JH. Independent prognostic value of coronary artery calcium score and coronary computed tomography angiography in an outpatient cohort of low to intermediate risk chest pain patients. Netherlands heart journal : monthly journal of the Netherlands Society of Cardiology and the Netherlands Heart Foundation. 2016;24(5):332-42. 10.1007/s12471-016-0819-5

7. Dedic A, Rossi A, Ten Kate GJ, Neefjes LA, Galema TW, Moelker A, et al. First-line evaluation of coronary artery disease with coronary calcium scanning or exercise electrocardiography. International journal of cardiology. 2013;163(2):190-5. 10.1016/j.ijcard.2011.06.002

8. Ergun E, Kosar P, Ozturk C, Basbay E, Koc F, Kosar U. Prevalence and extent of coronary artery disease determined by 64-slice CTA in patients with zero coronary calcium score. Int J Cardiovasc Imaging. 2011;27(3):451-8. 10.1007/s10554-010-9681-5

9. Gabriel FS, Goncalves LFG, Melo EV, Sousa ACS, Pinto IMF, Santana SMM, et al. Atherosclerotic Plaque in Patients with Zero Calcium Score at Coronary Computed Tomography Angiography. Arq Bras Cardiol. 2018;110(5):420-7. 10.5935/abc.20180063

10. Gottlieb I, Miller JM, Arbab-Zadeh A, Dewey M, Clouse ME, Sara L, et al. The absence of coronary calcification does not exclude obstructive coronary artery disease or the need for revascularization in patients referred for conventional coronary angiography. Journal of the American College of Cardiology. 2010;55(7):627-34. 10.1016/j.jacc.2009.07.072

11. Haberl R, Becker A, Leber A, Knez A, Becker C, Lang C, et al. Correlation of coronary calcification and angiographically documented stenoses in patients with suspected coronary artery disease: results of 1,764 patients. Journal of the American College of Cardiology. 2001;37(2):451-7. 10.1016/s0735-1097(00)01119-0

12. Hulten E, Bittencourt MS, Ghoshhajra B, O'Leary D, Christman MP, Blaha MJ, et al. Incremental prognostic value of coronary artery calcium score versus CT angiography among symptomatic patients without known coronary artery disease. Atherosclerosis. 2014;233(1):190-5. 10.1016/j.atherosclerosis.2013.12.029

13. Kelly JL, Thickman D, Abramson SD, Chen PR, Smazal SF, Fleishman MJ, et al. Coronary CT angiography findings in patients without coronary calcification. AJR Am J Roentgenol. 2008;191(1):50-5. 10.2214/AJR.07.2954

14. Kim YJ, Hur J, Lee HJ, Chang HJ, Nam JE, Hong YJ, et al. Meaning of zero coronary calcium score in symptomatic patients referred for coronary computed tomographic angiography. European heart journal cardiovascular Imaging. 2012;13(9):776-85. 10.1093/ehjci/jes060

15. Liu YC, Sun Z, Tsay PK, Chan T, Hsieh IC, Chen CC, et al. Significance of coronary calcification for prediction of coronary artery disease and cardiac events based on 64-slice coronary computed tomography angiography. BioMed research international. 2013;2013:472347. 10.1155/2013/472347

16. Meyer M, Henzler T, Fink C, Vliegenthart R, Barraza JM, Jr., Nance JW, Jr., et al. Impact of coronary calcium score on the prevalence of coronary artery stenosis on dual source CT coronary angiography in caucasian patients with an intermediate risk. Academic radiology. 2012;19(11):1316-23. 10.1016/j.acra.2012.06.006

17. Mittal TK, Pottle A, Nicol E, Barbir M, Ariff B, Mirsadraee S, et al. Prevalence of obstructive coronary artery disease and prognosis in patients with stable symptoms and a zero-coronary calcium score. European heart journal cardiovascular Imaging. 2017;18(8):922-9. 10.1093/ehjci/jex037

18. Moradi M, Varasteh E. Coronary atherosclerosis evaluation among Iranian patients with zero coronary calcium score in computed tomography coronary angiography. Advanced biomedical research. 2016;5:24. 10.4103/2277-9175.175920

19. Nieman K, Galema TW, Neefjes LA, Weustink AC, Musters P, Moelker AD, et al. Comparison of the value of coronary calcium detection to computed tomographic angiography and exercise testing in patients with chest pain. Am J Cardiol. 2009;104(11):1499-504. 10.1016/j.amjcard.2009.07.011

20. Parsons I, Pavitt C, Chamley R, d'Arcy J, Nicol E. CT Coronary Angiography vs. Coronary Artery Calcium Scoring for the Occupational Assessment of Military Aircrew. Aerospace medicine and human performance. 2017;88(2):76-81. 10.3357/amhp.4636.2017

21. Schuhbaeck A, Schmid J, Zimmer T, Muschiol G, Hell MM, Marwan M, et al. Influence of the coronary calcium score on the ability to rule out coronary artery stenoses by coronary CT angiography in patients with suspected coronary artery disease. Journal of cardiovascular computed tomography. 2016;10(5):343-50. 10.1016/j.jcct.2016.07.014

22. Sosnowski M, Pysz P, Szymanski L, Gola A, Tendera M. Negative calcium score and the presence of obstructive coronary lesions in patients with intermediate CAD probability. International journal of cardiology. 2011;148(1):e16-8. 10.1016/j.ijcard.2009.01.077

23. Ueda H, Harimoto K, Tomoyama S, Tamaru H, Miyawaki M, Mitsusada N, et al. Relation of cardiovascular risk factors and angina status to obstructive coronary artery disease according to categorical coronary artery calcium score. Heart Vessels. 2012;27(2):128-34. 10.1007/s00380-011-0128-2
